# Computational Tools and Resources for Long-read Metagenomic Sequencing Using Nanopore and PacBio

**DOI:** 10.1093/gpbjnl/qzaf075

**Published:** 2025-08-22

**Authors:** Tianyuan Zhang, Mian Jiang, Hanzhou Li, Yunyun Gao, Salsabeel Yousuf, Kaimin Yu, Xinxin Yi, Jun Wang, Lulu Yang, Yong-Xin Liu

**Affiliations:** Genome Analysis Laboratory of the Ministry of Agriculture and Rural Affairs, Agricultural Genomics Institute at Shenzhen, Chinese Academy of Agricultural Sciences, Shenzhen 518120, China; Wuhan Benagen Technology Co., Ltd., Wuhan 430000, China; Wuhan Benagen Technology Co., Ltd., Wuhan 430000, China; Wuhan Benagen Technology Co., Ltd., Wuhan 430000, China; Genome Analysis Laboratory of the Ministry of Agriculture and Rural Affairs, Agricultural Genomics Institute at Shenzhen, Chinese Academy of Agricultural Sciences, Shenzhen 518120, China; Genome Analysis Laboratory of the Ministry of Agriculture and Rural Affairs, Agricultural Genomics Institute at Shenzhen, Chinese Academy of Agricultural Sciences, Shenzhen 518120, China; Wuhan Benagen Technology Co., Ltd., Wuhan 430000, China; Wuhan Benagen Technology Co., Ltd., Wuhan 430000, China; Wuhan Benagen Technology Co., Ltd., Wuhan 430000, China; Wuhan Benagen Technology Co., Ltd., Wuhan 430000, China; Genome Analysis Laboratory of the Ministry of Agriculture and Rural Affairs, Agricultural Genomics Institute at Shenzhen, Chinese Academy of Agricultural Sciences, Shenzhen 518120, China

**Keywords:** Metagenome, Nanopore, PacBio, Software, Database

## Abstract

In recent years, the field of shotgun metagenomics has witnessed remarkable advancements, primarily driven by the development and refinement of next-generation sequencing technologies, particularly long-read sequencing platforms such as Nanopore and PacBio. These platforms have significantly improved the ability to analyze microbial communities directly from environmental samples, providing valuable information on their composition, function, and dynamics without the need for pure cultivation. These technologies enhance metagenomic data assembly, annotation, and analysis by addressing longer reads, higher error rates, and complex data. In this review, we provide a comprehensive overview of the historical development of long-read metagenomics, highlighting significant landmarks and advancements. We also explore the diverse applications of long-read metagenomics, emphasizing its impact across various fields. Additionally, we summarize the essential computational tools and resources, including software, databases, and packages, developed to enhance the efficiency and accuracy of metagenomic analysis. Finally, we provide a practical guide for the installation and use of notable software available on GitHub (https://github.com/zhangtianyuan666/LongMetagenome). Overall, this review assists the metagenomics community in exploring microbial life in unprecedented depth by providing a roadmap for successful resource utilization and emphasizing possibilities for innovation.

## Graphical abstract

**Figure:**
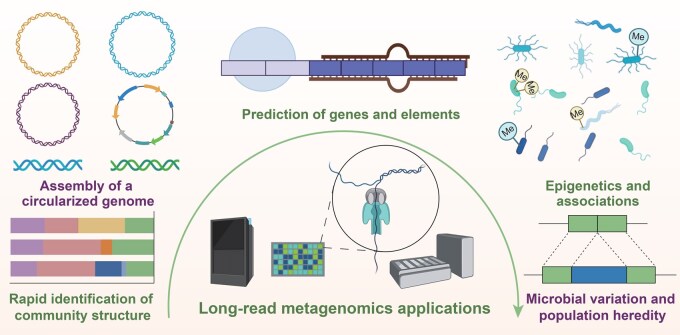

## Overview of the history of long-read metagenomics

**Figure 1:**
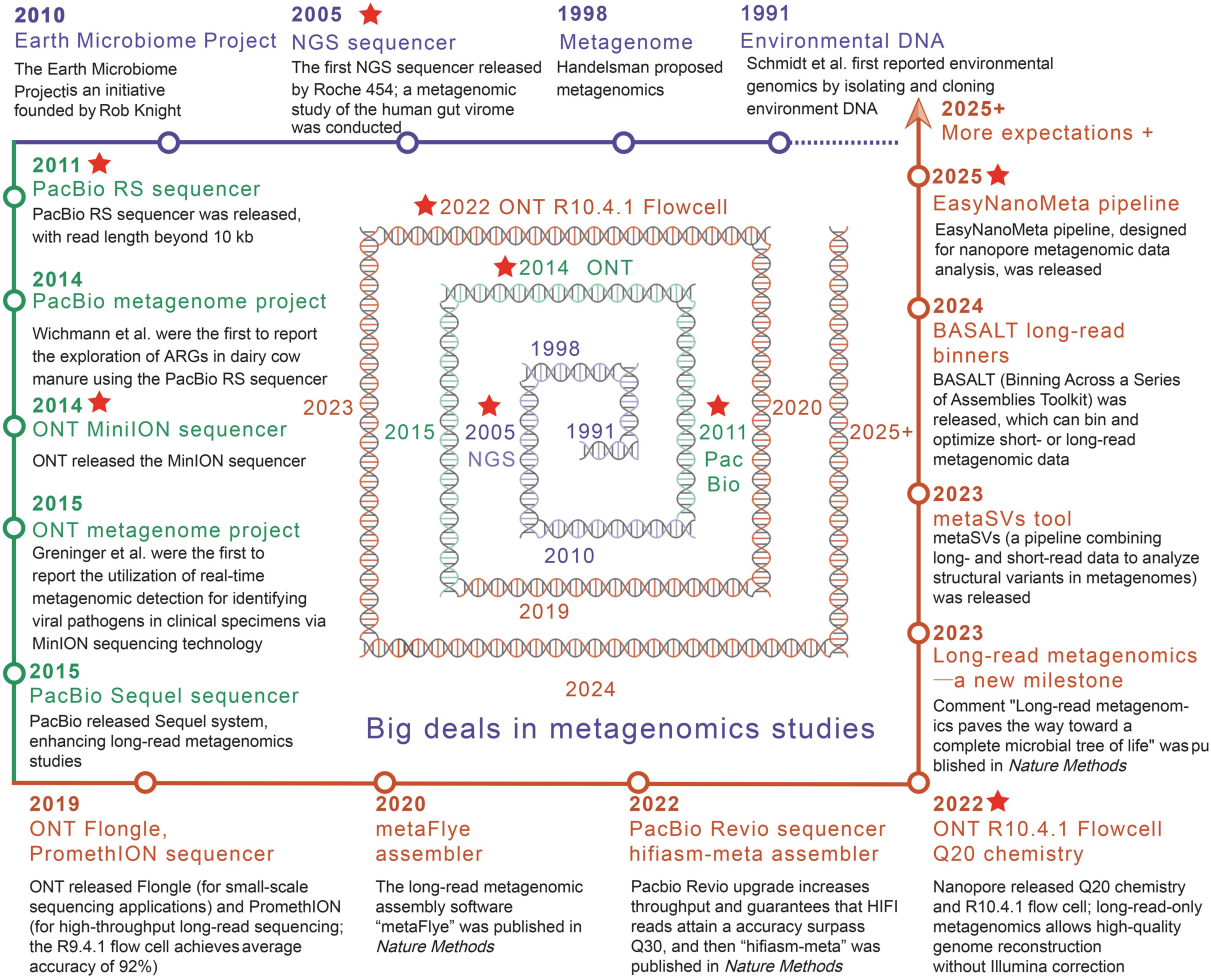
The origin and development of the long-read metagenomics studies Purple represents the origin stage of metagenomics, symbolizing the early beginnings and conception of the field (1991–2010). Green signifies the development of long-read metagenomics, indicating a period of advancement during which longer DNA sequencing reads were introduced, enhancing the resolution and capabilities of metagenomic analysis (2011–2018). Orange signifies the maturation and expansion phase of long-read metagenomics, highlighting a stage when this technology became more refined, widely adopted, and broadly applied (2019–2025+). NGS, next-generation sequencing; PacBio, Pacific Biosciences; ONT, Oxford Nanopore Technologies; ARG, antibiotic resistance gene.

Most microorganisms in nature are difficult to isolate and cultivate. The concept of metagenome, introduced by Handelman et al. in 1998, encompasses the genetic material from both cultivable and uncultivable microorganisms [1]. Over the past two decades, metagenomic technology has become a powerful tool for studying microbial communities, as it eliminates the need for cultivation [2–4]. Metagenomic research advanced significantly with the introduction of the first high-throughput sequencer in 2005. These sequencers can generate vast amount of sequence data from DNA simultaneously, enabling scientists to explore the genetic diversity of soil, water, human gut, and other environmental microbiota. This breakthrough has revealed numerous novel microbial species, genes, and metabolic pathways. Currently, metagenomic research has made significant advancements, expanding its potential applications (**Figure 1**). However, short-read shotgun metagenomic sequencing is widely used due to its ease and accessibility, but it has certain limitations in assembly, as well as detecting structural variations (SVs) and duplication regions [5,6]. Additionally, the inability to cover longer repeats and homologous regions restricts the capacity to differentiate polymorphic sites and distinguish between closely related species or strains. Consequently, analyzing the complex composition of microbial communities may not accurately distinguish species.

Long-read sequencing methods, such as Oxford Nanopore Technologies (ONT) and Pacific Biosciences (PacBio), have revolutionized genomics by enabling the generation of extraordinarily long DNA sequences. The PacBio RS sequencer, introduced in 2011, has the capacity to generate read lengths exceeding 10 kilobases (kb). Early PacBio sequencing, however, was characterized by a high error rate of around 11%–15% for long reads [7]. Despite this, PacBio technology was not applied to investigate resistance genes in the metagenome of dairy cow manure until 2014 [8]. In the same year, ONT released the MinION sequencer, achieving an accuracy of about 64% [9]. In 2015, Greninger et al. were the first to use real-time, unbiased metagenomic detection to identify viral infections in clinical specimens using MinION nanopore sequencing technology [10]. This marked the beginning of the long-read metagenomics era. Unlike short-read sequencing methods, which typically yield DNA fragments ranging from a few dozen to several hundred base pairs, long-read sequencing technologies can produce reads spanning thousands to tens of thousands of base pairs. This capability has profoundly impacted metagenomics, enabling the study of microbial communities in environmental samples and providing a comprehensive and accurate understanding of these complex ecosystems.

Advancements in sequencing technology have significantly reduced the error rates of PacBio and ONT platforms. PacBio has released the Sequel sequencers, which utilize circular consensus sequencing (CCS) mode to produce high-fidelity (HiFi) reads with an accuracy of quality score 20 (Q20; **Box 1**) or higher. In 2019, ONT launched the portable sequencer Flongle and the commercial high-throughput sequencer PromethION, significantly increasing the application of long-read metagenomes. Flongle and MinION support real-time sequencing and analyses in field environments, including outdoor sites and even on Earth’s space stations [11]. Meanwhile, advancements in the chemistry of the R9.4.1 flow cell (Box 1) have enabled an average accuracy of 92% [12]. Additionally, the first long-read metagenomic assembly software “metaFlye” has been published, demonstrating excellent performance [13,14]. By 2022, the R10.4.1 flow cells equipped with Q20+ chemistry were capable of generating data with an accuracy of ≥ Q20. Besides, the upgrade to the PacBio Revio sequencer enhanced throughput, reduced sequencing time to 24 h, and ensured that HiFi reads attained an accuracy surpassing Q30, reflecting an exceptional level of accuracy in long-read sequencing. Following these advancements, the “HiFiasm-meta” assembler was introduced for HiFi metagenome assembly. In January 2023, the comment titled “Long-read metagenomics paves the way toward a complete microbial tree of life” was published in the prestigious journal *Nature Methods*, representing a notable achievement in the field of long-read metagenomics [15]. This insightful comment predicted that long-read sequencing technologies would progressively unveil the complexity and diversity of the microbial world, ultimately enabling a complete microbial tree of life. Since then, the application of long-read metagenomic technology has steadily advanced, with more researchers employing these approaches in their research. For instance, Huang et al. combined ONT and PacBio long-read sequencing with Illumina short-read sequencing to establish a high-quality Panda gut microbiome catalog (pandaGUT) [16]. Presently, tools and resources specifically designed for long-read metagenomes are being developed, including software for identifying and classifying SVs in metagenomic data, such as metaSVs [17] and the latest binning (Box 1) software BASALT [18]. The new Chinese nanopore platform CycloneSEQ generated 7.7 gigabase (Gb) of long-read data from the ZymoBIOMICS Gut Microbiome Standard mock sample. It effectively quantified the relative DNA abundance of 15 species and successfully assembled genomes for 10 species with over 1% abundance, 9 of which were circularized [19]. In March 2025, a comprehensive evaluation of existing tools for nanopore-based metagenomic analysis was performed, leading to the development of an integrated bioinformatics pipeline, EasyNanoMeta [20], designed to address challenges in analyzing nanopore-based metagenomic data. These advancements underscore the growing importance of long-read sequencing in metagenomics, enabling more accurate microbial analyses and driving novel discoveries.

Subsequently, we will review and introduce the latest software, databases, and bioinformatics pipelines that are applicable to long-read metagenomics studies and applications.

## Applying long-read metagenomics to analyze microbial community structure and functions

**Figure 2:**
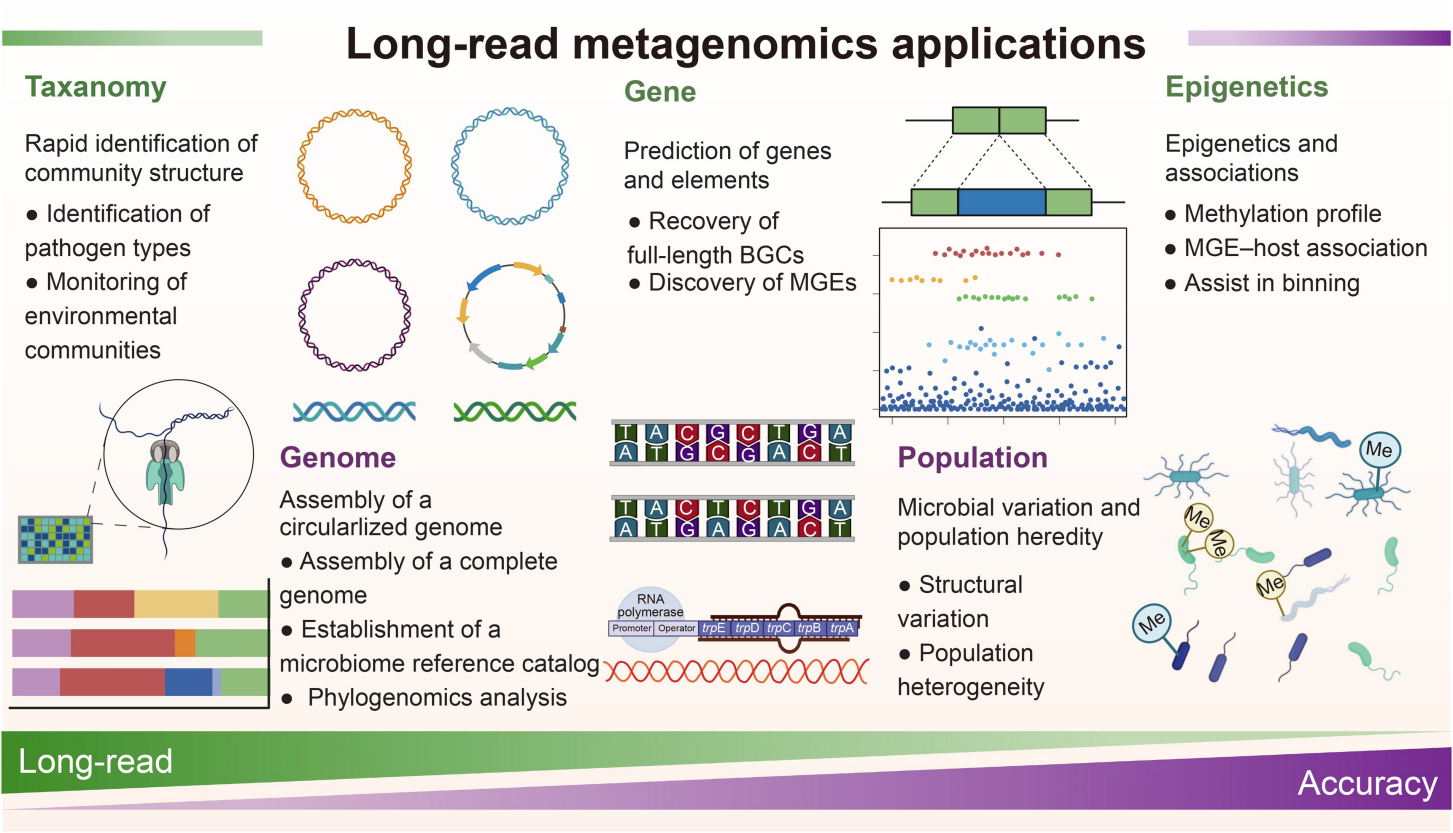
Applications of long-read metagenomics in analyzing microbial community structure and functions BGC, biosynthetic gene cluster; MGE, mobile genetic element.

By providing continuous sequences that can cover entire genes, operons, or even genomes, long-read metagenomic sequencing has greatly improved microbial community analysis [21,22]. Unlike short-read sequencing methods, long-read sequencing offers comprehensive insights into the functional potential of complex microbial communities, overcoming previous limitations. Consequently, it has gained popularity among researchers. In this review, we examine the long-read metagenomics applications from the past decade (**Figure 2**).

### Rapid identification of community structure

Long-read sequencing can detect rare or low-abundance species that short-read sequencing cannot, providing a more comprehensive profile of microbial diversity within a sample. This enhanced resolution is essential for comprehending the structure and function of microbial communities across diverse environments, including soil, water, and the human gastrointestinal tract. ONT, in particular, has facilitated real-time sequencing and analysis, enabling rapid pathogen identification [23,24] and *in situ* monitoring of environmental communities [25].

### Assembly of a circularized genome

In addition, long-read sequencing data facilitate the assembly of continuous genomic sequences by overcoming repeat regions and SVs, distinguishing it from short-read assembly methods. Thus, long-read data can be handled more efficiently through assembly and binning to obtain the complete genome [26]. Furthermore, it supports the development of microbiome reference catalogs [16,27] and the investigation of phylogenomic relationships between closely related genomes [28].

### Prediction of genes and elements

The majority of antibiotics and drugs used in clinical settings are derived from natural compounds found in plants or microbes. Integrating traditional separation and analysis approach with metagenomic mining simplifies the identification and characterization of natural product pathways based on genomic data. Moreover, it enables the recovery of complete biosynthetic gene cluster (BGC) sequences and identification of novel biosynthetic pathways for drug development [29]. It also aids in uncovering mobile genetic elements (MGEs), such as antibiotic resistance genes (ARGs) [30,31] and metal resistance genes (MRGs) [32]. Long-read sequencing reveals insights into microbial communities, as well as the diversity and relationships of MGEs. MGEs, including plasmids, transposons, and bacteriophages, promote horizontal gene transfer (HGT) between microbial species, significantly speeding up evolutionary dynamics and adaptive responses. Long-read sequencing covers and characterizes multiple MGE and HGT events, revealing mechanisms of microbial evolution and community composition.

### Microbial variation and population heredity

Investigating the diversity of microorganisms within a population is crucial for comprehending microbial ecology, evolution, and their influence on human health. Long-read sequencing data, which span complex genomic regions, facilitate the identification of SVs, such as insertions, deletions, inversions, and translocations that may be overlooked by short-read sequencing techniques [33]. Hence, long-read data provide access to a variety of SVs and enable the quantification of population heterogeneity in metagenomics [17,34,35].

### Epigenetics and associations

PacBio sequencing technology detects single-base methylation by leveraging its unique real-time fluorescence signal to monitor base insertion. Similarly, nanopore sequencing can detect base modifications, including epigenetic signatures such as 5-methylcytosine (5mC) and N^6^-methyladenine (6mA). In metagenomic epigenetics, both PacBio and ONT data can be utilized for DNA methylation analysis, helping characterize different bacterial species within the metagenome [36,37]. Additionally, this approach offers a method for evaluating microbial genomes with unusual size and structural complexity from metagenomes [38]. Furthermore, long-read metagenomics combined with Hi-C or metaPore-C technology provide linkage information between plasmids, hosts, and viruses [39–41].

## Software, databases, and downstream tools for long-read metagenomics

### Description and evaluation of software for long-read metagenomic analysis

**Figure 3:**
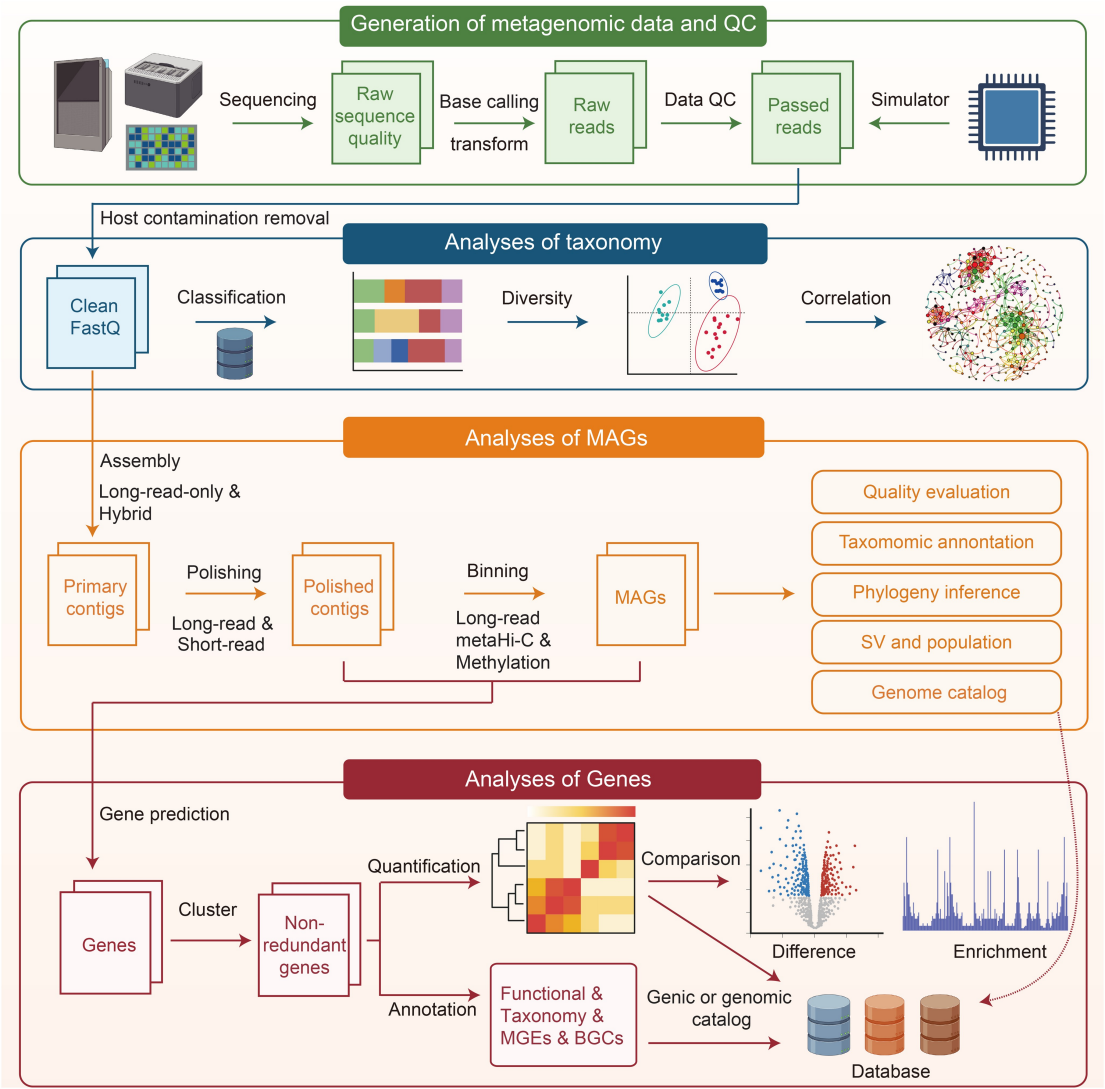
Bioinformatics pipeline for long-read metagenomic data analysis QC, quality control; MAG, metagenome-assembled genome; SV, structural variation; Hi-C, high-resolution chromosome conformation capture.

The applications of long-read metagenomics are rapidly expanding, providing substantial data that drive improvement in computational models for analysis. Therefore, we summarize bioinformatics pipelines applicable to long-read metagenomic analysis (**Figure 3**). **Table 1** presents popular tools, while Table S1 provides a comprehensive list and overview of currently available tools for long-read metagenomic analysis.

**Table 1:** Noteworthy software in metagenomics studies

Software	Description	Website	Ref.
**Data quality control, simulator, and host removal**			
SMRTlink^a^	PacBio official workflows ranging from base calling to sequence alignment	https://www.pacb.com/support/software-downloads/	–
bam2fastx	Converting BAM-formatted sequencing data to FASTQ format	https://github.com/PacificBiosciences/bam2fastx	[42]
Dorado^a^	A newer base-calling tool to replace Guppy	https://github.com/nanoporetech/dorado	–
PBSIM3^a^	A simulator for all types of PacBio and ONT long reads	https://github.com/yukiteruono/pbsim3	[43]
Porechop^a^	Adapter and chimera trimmer for Oxford Nanopore reads	https://github.com/rrwick/Porechop	–
NanoFilt^a^	Filtering and trimming of nanopore long reads	https://github.com/wdecoster/nanofilt	[46]
LongQC^a^	Quality control of PacBio and ONT long reads	https://github.com/yfukasawa/LongQC	[47]
Minimap2^a^	A versatile pairwise aligner for long reads	https://github.com/lh3/minimap2	[49]
Winnowmap2^a^	Long-read or genome alignment software based on Minimap2	https://github.com/marbl/Winnowmap	[50]
LAST^a^	Pairwise genome alignments	https://gitlab.com/mcfrith/last	[62]
**Taxonomy profiling and read binning**			
Kraken2	K-mer based taxonomic classifier	https://ccb.jhu.edu/software/kraken2	[54]
Bracken	Bayesian estimation of abundance with Kraken	https://ccb.jhu.edu/software/bracken/	[56]
BugSeq^a^	Alignment, LCA algorithm, and a cloud platform for long-read metagenomics	https://bugseq.com/free	[60]
Metamaps^a^	Mapping algorithm and expectation-maximization-based estimation for long-read metagenomic analysis	https://github.com/DiltheyLab/MetaMaps	[61]
MEGAN-LR^a^	Alignment and LCA algorithm for taxonomic binning	http://ab.inf.uni-tuebingen.de/software/downloads/megan-lr	[63]
deSAMBA^a^	A tailored long-read classifier	https://github.com/hitbc/deSAMBA	[59]
Melon^a^	Taxonomic identification and quantification in long-read metagenomics using marker genes	https://github.com/xinehc/melon	[64]
Diamond	Sequence aligner for protein and translated DNA searches, faster than BLAST	https://github.com/bbuchfink/diamond	[65]
metaBCC-LR^a^	Long-read binner with K-mer, composition, and density-based clustering	https://github.com/anuradhawick/MetaBCC-LR	[67]
LRBinner^a^	Long-read binner with K-mer and latent representation	https://github.com/anuradhawick/LRBinner	[68]
**Metagenome assembly, polishing, and binning**			
HiFiasm-meta^a^	Haplotype-resolved assembler for accurate HiFi reads	https://github.com/lh3/HiFiasm-meta	[70]
metaFlye^a^	*De novo* assembler for long reads using repeat graphs	https://github.com/fenderglass/Flye	[14]
Lathe^a^	Generating bacterial genomes from metagenomes with nanopore sequencing	https://github.com/bhattlab/lathe	[26]
metaMDBG^a^	Assembler for long and accurate metagenomics reads (*e.g.*, PacBio HiFi) based on the MDBG	https://github.com/GaetanBenoitDev/metaMDBG	[71]
STRONG^a^	Metagenomic strain resolution on assembly graphs	https://github.com/chrisquince/STRONG	[72]
Strainberry^a^	Automated strain separation of low-complexity metagenomes	https://github.com/rvicedomini/strainberry	[73]
OPERA-MS^a^	Hybrid metagenomic assembler which combines short and long reads	https://github.com/CSB5/OPERA-MS	[74]
Pilon	Improving assemblies by correcting bases, fixing misassemblies, and filling gaps via hierarchical polishing	https://github.com/broadinstitute/pilon	[76]
Racon^a^	Standalone consensus module to correct raw contigs via partial order alignment graph	https://github.com/isovic/racon	[77]
Medaka^a^	Correcting draft sequences, and creating consensus sequences and variant calls from nanopore sequencing data via neural network model	https://github.com/nanoporetech/medaka	–
Ratatosk^a^	Hybrid error correction of long reads using colored de Bruijn graphs	https://github.com/DecodeGenetics/Ratatosk	[79]
MetaBAT2	Similarity-based binner with label propagation algorithm	https://bitbucket.org/berkeleylab/metabat	[81]
metaWRAP	Similarity-based binner with ensemble learning, integrating MetaBAT2, MaxBin2, and Concoct	https://github.com/bxlab/metaWRAP	[82]
GraphMB^a^	Long-read binner with graph neural networks that integrates the assembly graph into binning	https://github.com/MicrobialDarkMatter/GraphMB	[84]
MetaCoAG^a^	Short- and long-read binner via composition, coverage, and assembly graphs	https://github.com/metagentools/MetaCoAG	[85]
MUFFIN^a^	Hybrid assembly and binning workflow for metagenomics, transcriptomics, and pathway analysis	https://github.com/RVanDamme/MUFFIN	[87]
BASALT^a^	Binning Across a Series of Assemblies Toolkit for short and long reads	https://github.com/EMBL-PKU/BASALT	[18]
HiCBin	Binning using Hi-C contact maps	https://github.com/dyxstat/HiCBin	[88]
MetaCC^a^	Binning long- and short-read metagenomic Hi-C data	https://github.com/dyxstat/MetaCC	[40]
Nanodisco^a^	Discovering multiple types of DNA methylation, and binning using nanopore sequencing	https://github.com/fanglab/nanodisco	[36]
dRep	Rapidly comparing large numbers of genomes and choosing the best representative genome	https://github.com/MrOlm/drep	[90]
GTDB-tk	Taxonomic classifications for bacterial and archaeal genomes	https://ecogenomics.github.io/GTDBTk/	[91]
Bugsplit	Highly accurate taxonomic binning of metagenomic assemblies	https://bugseq.com/academic	[92]
CheckM2	Predicting the completeness and contamination of genomic bins using machine learning	https://github.com/chklovski/CheckM2	[93]
CoverM	Calculating coverage of genomes/MAGs	https://github.com/wwood/CoverM	[94]
metaQUAST	Evaluation of metagenome assemblies	http://bioinf.spbau.ru/metaquast	[95]
MetaCortex	Capturing variations in metagenomic assembly graphs	https://github.com/SR-Martin/metacortex	[96]
StrainPhlAn	Profiling microbes from known species with strain-level resolution and providing comparative and phylogenetic analysis	http://segatalab.cibio.unitn.it/tools/strainphlan/	[97]
Strainy^a^	Phasing and assembly of strain haplotypes using long-read data	https://github.com/katerinakazantseva/strainy	[98]
MAGphase^a^	Phasing for metagenomics using PacBio long-read data	https://github.com/Magdoll/MagPhase	[99]
metaSVs^a^	Combining long- and short-read data for analysis and visualization of structural variants in metagenomes	https://github.com/Wlab518/SV_procedure	[17]
**Gene prediction and functional analysis**			
Prokka	Rapid prokaryotic genome annotation	https://github.com/tseemann/prokka	[100]
HMMER	Searching sequence databases for sequence homologs by HMMs	http://hmmer.org/	[106]
BLAST+	Finding regions of similarity between biological sequences	https://blast.ncbi.nlm.nih.gov/Blast.cgi	[107]
eggNOG-mapper	Functional annotation of novel sequences from the eggNOG database	http://eggnog-mapper.embl.de/	[113]
antiSMASH	Searching a genome sequence for secondary metabolite BGCs	https://antismash.secondarymetabolites.org/	[114]
BiG-SCAPE	Constructing sequence similarity networks of BGCs and grouping them into cluster families	https://bigscape-corason.secondarymetabolites.org/	[115]
PlasFlow	Prediction of plasmid sequences in metagenomic contigs	https://github.com/smaegol/PlasFlow	[116]
PhiSpy	Finding prophages in bacterial genomes that combines similarity- and composition-based strategies	https://github.com/linsalrob/PhiSpy	[117]
Salmon	Highly-accurate, transcript-level quantification tools suitable for metagenomic data	https://github.com/COMBINE-lab/salmon	[118]
Cd-hit	Clustering and comparing protein or nucleotide sequences	https://github.com/weizhongli/cdhit	[112]

*Note*: ^a^, the software developed for long-read metagenomics. The installation and usage methods for noteworthy software have been uploaded to GitHub (https://github.com/zhangtianyuan666/LongMetagenome). This table will be updated on a quarterly basis, taking into account developments in the field, reader feedback, and the extent of maintenance and updates carried out by the authors. PacBio, Pacific Biosciences; ONT, Oxford Nanopore Technologies; LCA, lower common ancestor; deSAMBA, de Bruijn graph-based Sparse Approximate Match Block Analyzer; HiFi, high-fidelity; MDBG, minimizer de-Brujin graph; MAG, metagenome-assembled genome; BLAST, Basic Local Alignment Search Tool; BGC, biosynthetic gene cluster; HMM, hidden Markov model.

#### Data quality control, simulator, and host removal

PacBio data need to be analyzed after sequencing is completed. The raw PacBio data must be processed and analyzed using SMRTlink (https://www.pacb.com/support/software-downloads/), which manages the data and addresses issues such as linkers and low-quality reads. The bam2fastx [42] tool enables conversion of PacBio BAM files into FASTA and FASTQ files, with the capability to split barcoded data. Since 2023, ONT data analysis has benefited from the Dorado basecaller (https://github.com/nanoporetech/dorado). For simulating various types of PacBio and ONT long-read data, tools like PBSIM3 [43], Meta-NanoSim [44], and PaSS [45] are employed. Quality control steps include using Porechop (https://github.com/rrwick/Porechop) to demultiplex ONT reads, identify barcodes, trim adapter sequences, and remove potential chimeric reads, thereby improving data quality for downstream analyses. Additionally, NanoFilt [46] filters and trims ONT reads based on quality, length, and adapter, promoting high-quality data preprocessing, and it can also be applied to PacBio data. LongQC [47] and Seqkit2 [48] can assess and confirm the quality of both Nanopore and PacBio data. These tools offer a range of functions, including quality assessment, read trimming, randomization of reads, and other essential functions for data preprocessing.

Consequently, it is essential to filter and remove host sequences by aligning them with a reference database of known host genomes. Long-read aligners are distinct from short-read aligners due to their specific capability to manage uninterrupted but error-prone sequences. These aligners require flexible algorithms to accommodate insertions and deletions (indels) while accurately navigating complex genomic regions. Among these tools, Minimap2 [49] has become one of the most widely utilized alignment tools for long-read data. Building on the Minimap2 framework, Winnowmap2 [50] introduces advanced features by combining a sophisticated seed search strategy with a semi-global alignment algorithm, making it particularly effective in handling long-read sequences with high error rates. Additionally, LAST [51] employs a global optimal alignment approach, offering rapid performance and high accuracy across various data types. Other long-read aligners, such as WFA-GPU [52] and BLASR [53], are also available, each contributing to enhanced precision and faster removal of host sequences.

#### Taxonomy profiling and read binning

The k-mer approach is commonly applied to process short-read data due to its efficiency and speed, making it ideal for handling large-scale datasets. It has also been employed in the analysis of long-read metagenomic data, leveraging inherent advantages such as scalability and providing rapid taxonomic assignment capabilities. Notably, Kraken2 [54] is renowned for its exceptional speed and is accompanied by a comprehensive suite of downstream processing tools, including KrakenTools [55] and Bracken [56]. FUNpore [57] addresses frameshift errors in nanopore reads and classifies these reads using Kraken. In addition, a few of long-read studies have employed Centrifuge [58] for taxonomy profiling. Many short-read tools are more susceptible to sequencing errors [59]. Conversely, alignment-based methods are preferred for long-read data, as they effectively utilize complete sequence information. BugSeq [60], Metamaps [61], LAST [62], MEGAN-LR [63], and Minimap2 [49] are among the prominent tools for long-read taxonomy profiling. BugSeq and Metamaps were developed to achieve strain-level resolution, with BugSeq being particularly notable for its faster analysis compared to Metamaps. Metamaps, the most popular tool, is recommended for its ability to use the National Center for Biotechnology Information (NCBI) Reference Sequence (RefSeq) database and apply the expectation-maximization (EM) algorithm to estimate species or strain-level abundance. It boasts several advantages, including rapid processing speed and low memory consumption. deSAMBA [59], a tool developed for long-read data, has not yet been widely adopted. A recently developed tool, Melon, designed for long-read metagenomic taxonomy profiling using marker genes, features an EM-based post-correction module that resolves ambiguous reads. It has demonstrated strong performance in both mock communities and wastewater samples [64]. Melon supports species classification using both the NCBI and Genome Taxonomy Database (GTDB) databases and is designed for easy installation. Other tools, such as Diamond [65] and Kaiju [66], are primarily developed for short-read data and are used for translation alignment to annotate the relative abundance of microbial species. Overall, Metamaps demonstrates superior performance compared to other tools. MetaBCC-LR [67] is a binner based on k-mer coverage that uses the DBSCAN algorithm, though its installation process can be challenging (requiring GCC v9.4.0). LRBinner [68], developed to improve the accuracy of binning long-read metagenomic data, utilizes k-mer profiles and variational autoencoder (latent representation) deep learning algorithms to combine composition and coverage information. LRBinner supports both read and contig binning, indicating superior performance compared to other tools while requiring less memory. Finally, MetaProb2 [69] uses minimizers for efficient read assembly into unitigs and applies a graph modularity-based community detection approach for clustering and identifying representative unitigs through an unsupervised binning method, using probabilistic k-mer statistics. However, it has not been updated since 2021.

#### Metagenome assembly, polishing, and binning

The metagenome comprises the genomes of numerous species, which often contains a significant abundance of repeat sequences both within and between species. During the assembly process, challenges arise from variations in read length distribution, high ploidy, and insufficient coverage of specific haplotypes. Long-read metagenomic sequencing significantly enhances sequence contiguity, reduces assembly ambiguity, improves genomic resolution in complex structures and repetitive regions, and facilitates genome assembly. HiFiasm-meta [70], designed for high-accuracy metagenomic data generated by PacBio HiFi sequencing, excels at handling complex metagenome assemblies with both high accuracy and efficiency, although it requires a substantial amount of memory. This tool allows for the potential reuse of units in multiple contigs and the assembly of circular genome sequences. While HiFiasm-meta may consume more resources and run slower, its precision in assembling complex microbial communities justifies these requirements. metaFlye [14], the most popular software for long-read metagenomic analysis, utilizes a repeat graph as its core data structure, effectively addressing the challenges of uneven bacterial composition in complex microbial communities and improving the integrity of assembly results. It supports several types of PacBio and ONT data with different error rates. Additionally, it offers a haplotype mode, which enables the identification of more heterozygous SVs. Lathe [26] combines long-read assembly and circularization approaches, utilizing the Flye assembler. This workflow, designed for long-read data from both ONT and PacBio technologies, produces high-quality circular genome assembly. Additionally, this workflow is encapsulated within the Snakemake framework, allowing researchers to adjust parameters for different community types. Its distinguishing features include support for short-read correction and genome circularization, though the program has not been updated since February 2021. metaMDBG [71], a new PacBio HiFi metagenomics assembler, employs de Bruijn graph assembly in a minimizer space with an iterative algorithm to handle uneven coverage depths across genomes. Additionally, the software now supports nanopore sequencing data. metaMDBG is particularly well-suited for handling repeated sequences and complex genomic regions, especially in situations with limited computational resources, due to its rapid execution. However, HiFiasm-meta offers superior accuracy when handling complex microbial communities. Other long-read metagenomic assembly tools include STRONG [72] and Strainberry [73]. In summary, for PacBio data, we recommend metaMDBG for speed and HiFiasm-meta for accuracy. For ONT data, we recommend Flye for faster performance and Lathe for a more comprehensive analysis.

Hybrid assembly, which combines long- and short-read data, is a commonly used approach for metagenome assembly. The most widely used tool for this approach is OPERA-MS [74], which follows a step-by-step process to assemble data, integrating findings from short-read assembly to successfully incorporate long-read data. By combining the high precision of short-read data with the extended coverage of long-read data, hybrid assembly enhances genome assembly outcomes, producing exceptional results. However, hybrid assembly software, such as hybridSPAdes [75], is developed based on single-genome assemblies and has restricted applications in metagenomics.

Given the various error rates of long-read data, it is essential to correct them after assembly. Pilon [76] is a widely used tool for improving and correcting long-read assemblies utilizing short-read data. For long-read-based polishing, several tools have been developed, especially for Nanopore data, though they can also be applied to PacBio data. Racon [77] and Medaka (https://github.com/nanoporetech/medaka) are most commonly used tools for guidelines. Notably, Racon employs efficient alignment techniques for rapid error correction and supports both ONT and PacBio data. In contrast, Medaka utilizes a deep learning model, is designed specifically for ONT data, and is not applicable to PacBio data. HiFi sequences provide high precision, making error correction optional. However, error correction is essential for ONT assemblies. Other tools like Nextpolish2 [78] and Homopolish [12] have also demonstrated efficacy in single-genome assemblies. Ratatosk [79] is a hybrid error correction tool for long-read data and assemblies, utilizing both long- and short-read sequences. Consequently, some researchers use short-read data to fix errors in long-read data before employing the corrected long-read data for assembly.

Metagenomic binning is a process of categorizing reads/contigs into groups, known as bins, according to shared attributes such as sequence composition, coverage, and taxonomic classification. Determining and analyzing specific taxonomic groups or genomes within complex microbial communities requires this process. Despite the challenges of binning long-read data due to a lack of information such as coverage and error rates [80], many tools based on short-read metagenomic partitioning are still widely used for long-read data. MetaBAT2 [81] is the most popular tool designed for short-read data and has been extensively employed in numerous studies involving long-read metagenomics. Empirically, MetaBAT2 is suitable for studies using short-read binning before long-read assembly. In addition, there are some binning tools based on short-read data, such as metaWRAP [82] and DAS_Tool [83], which are often used for comparison with newly developed tools. Recently, many long-read tools have been developed. GraphMB [84] and MetaCoAG [85] are novel binners that integrate advanced algorithms for long-read assemblies. GraphMB uses deep learning techniques in conjunction with the metaFlye assembly graph. Recent studies have shown that GraphMB performs exceptionally well on multiple gut sample datasets [86]. MetaCoAG [85] uses single-copy marker genes along with graph matching and label propagation algorithms to bin contigs generated by metaSPAdes, MEGAHIT, and Flye assemblies. This software also requires abundance calculations from CoverM and is notable for being the first fully autonomous contig-binning software, though its performance has yet to be widely validated. MUFFIN [87] is a comprehensive metagenomic workflow designed for the assembly, binning, and annotation of metagenomic data using both long- and short-read technologies. The integrated workflow offers a hybrid assembly approach and differential binning for metagenomics, transcriptomics, and pathway analysis. BASALT [18], a newly versatile tool, performs rapid binning and refinement of both short- and long-read data. It generates high-quality metagenome-assembled genomes (MAGs; Box 1) from PacBio, ONT, short-read, hybrid assembly, and Hi-C data by utilizing several binning tools and neural networks. We recommend using BASALT, which performs better than other tools, although it consumes more resources.

Metagenomic Hi-C (metaHi-C) is a 3D epigenomic technique used to detect links between contigs based on their physical proximity, making it highly useful for contig binning. Most metaHi-C analysis tools, such as HiCBin [88] and bin3C [89], were developed for short-read libraries. Although they can now process long-read data, there remains potential to improve their efficacy. MetaCC [40] provides outstanding efficiency compared to current tools, excelling in the analysis of both long- and short-read data with metaHi-C. Moreover, while PacBio and ONT are primarily recognized for generating ultra-long-read data, they also provide valuable data for investigating epigenetic information. Therefore, we recommend using metaCC as it outperforms other tools. The Nanodisco [36] toolbox employs nanopore sequencing to discover all three types of DNA methylation (6mA, 5mC, and N^4^-methylcytosine) across bacterial genomes and microbiomes. It also uses these distinct epigenetic patterns to perform high-resolution metagenomic binning on microbiome samples.

The subsequent step involves the downstream analysis of MAGs. Many software tools originally designed for short-read metagenomes are also adaptable to long-read data. For example, dRep [90] is an efficient genome dereplication tool that clusters MAGs based on nucleotide similarity, facilitating the identification of distinct genomic entities and reducing redundancy in genomic datasets. For the taxonomy of MAGs, GTDB-tk [91] is highly effective in classifying bacteria and archaea, even when dealing with large numbers of genomes. Bugsplit [92] categorizes MAGs based on taxonomy using a reference database, highlighting the ability of long-read data to automate the identification of microorganisms in complex microbial communities. To assess the quality of MAGs, CheckM2 [93] is commonly used for assessing completeness and contamination, while CoverM [94] provides advanced analyses of metagenomic datasets, specifically assessing MAG coverage and completeness. MetaQUAST [95] evaluates the quality of metagenome assemblies by computing misassemblies, unaligned contigs, and gene predictions.

Several tools have been developed to quantify and categorize the diversity within a species using metagenomic data. MetaCortex [96] identifies differences by analyzing polymorphism symbols, providing insight into minor variations such as single nucleotide polymorphisms (SNPs) and indels. StrainPhlAn [97] utilizes single nucleotide variants (SNVs) in marker genes to categorize internal variations of species into clusters, which is extremely effective for phylogenetic reconstruction and population genetic studies of uncultivated or unidentified species. A recently developed tool, Strainy [98], is designed for phasing and assembling strain haplotypes using long-read data. It takes a *de novo* metagenomic assembly as input to identify strain variants. It constructs a connection graph for each strain-collapsed contig, encoding the pairwise distances between aligned reads. The reads are then clustered by strain using community detection, and this clustering is refined with increased sensitivity to strain variants, allowing for the separation of closely related strains and their assembly into contiguous haplotypes. MAGphase [99] is designed for phasing metagenomic assembly graphs using PacBio reads, enabling the identification of genomic SNP haplotypes within metagenomic datasets. metaSVs [17] applies both nanopore long- and short-read data to investigate SVs among complex microbial communities.

#### Gene prediction and functional analysis

By incorporating structural and functional annotation outcomes, one can gain an understanding of the potential functions and biological significance of metagenomic data. Following the process of sequence assembly or binning, gene prediction becomes an essential step in genome annotation. Gene prediction tools identify genomic DNA regions that encode genes, including regulatory elements, protein-coding genes, and RNA genes. Because of the annotation of MAGs, most software based on short-read metagenomes is also suitable for long-read data. Prokka [100] is a powerful command-line tool for annotating prokaryotic genomes, especially those of bacteria and archaea. To accomplish comprehensive genome annotation, Prodigal [101] is employed for gene prediction, Aragorn [102] for tRNA prediction, and Barrnap (https://github.com/tseemann/barrnap) for ribosomal RNA (rRNA) identification. Furthermore, MetaGeneMark2 [103], NCBI-PGAP [104], and Glimmer-MG [105] are also employed for gene prediction. To ensure thorough and accurate annotation, tools like HMMER [106] are used to identify protein domains, while BLAST+ [107] assists in searching databases like UniProt [108] for homologous proteins. Other tools include tRNAscan-SE [109] for tRNA identification, Minced [110] for CRISPR recognition, DeepTMHMM [111] for predicting signal peptides and transmembrane domains. Likewise, CD-HIT [112] is widely used for creating non-redundant gene or protein sequences.

Additionally, functional annotation tools provide valuable insights. EggNOG-mapper [113] swiftly maps protein sequences to orthologous groups using the eggNOG database, offering functional annotations, phylogenetic insights, and protein domain compositions. This method is particularly valuable for newly sequenced organisms with limited annotations, as it predicts unknown proteins based on functional and evolutionary data. BGCs comprise enzymes and regulatory factors responsible for producing secondary metabolites. AntiSMASH 6.0 [114] automatically identifies and annotates BGCs in MAGs. When combined with BiG-SCAPE [115], researchers can streamline the exploration of natural product biosynthesis pathways, facilitating the identification of novel pathways. Plasmids, self-replicating entities within prokaryotic cells, play a crucial role in genetic diversity and evolution. PlasFlow [116] efficiently identifies plasmid sequences in genomic and metagenomic data. Phispy [117] is another tool capable of identifying active prophages, contributing to a better understanding of viral elements within microbial genomes. Salmon [118] is also a widely used tool that offers rapid and unbiased quantification of gene expression.

### The databases in long-read metagenomics studies

In the field of long-read metagenomics, databases play a crucial role in analyzing and interpreting the massive datasets generated from various microbial communities. As most database analyses focus on contig- or gene-level assessments, these databases are suitable for both general and long-read metagenomics studies. Below is an overview of the primary databases utilized in metagenomics (**Table 2**).

**Table 2:** Databases in metagenomics studies

Database	Description	Tool	Website	Ref.
**Functional annotation / reference databases**				
Nr	NCBI non-redundant database	BLAST+	https://ftp.ncbi.nlm.nih.gov/blast/db/FASTA/	[119]
UniProt	Database of protein sequence and functional information for all species	BLAST+	https://www.uniprot.org/	[123]
GO	The Gene Ontology focuses on the function of the genes and gene products	BLAST+, BLAST2GO	https://www.geneontology.org/	[122]
KEGG	Kyoto Encyclopedia of Genes and Genomes	Kofamscan, BLAST+, KOBAS	https://www.genome.jp/kegg/	[124]
Nt	NCBI nucleotide database	BLAST+	https://www.ncbi.nlm.nih.gov/nucleotide/	[120]
RefSeq	NCBI reference sequence database	BLAST+	https://www.ncbi.nlm.nih.gov/refseq/	[121]
EggNOG	Ortholog linkages, functional annotations, and gene evolutionary	EggNOG-mapper	http://eggnog5.embl.de/	[113]
Rfam	RNA families database	HMMER	https://rfam.org/	[125]
TIGRFAMs	Inferring protein families and domains based on HMMs	HMMER	https://www.tigr.org/TIGRFAMs	[127]
MBGD	Microbial genome database for comparative analysis	BLAST+	https://mbgd.nibb.ac.jp/	[128]
**Resistance and mobile genetic element databases**				
mobileOG-db	Bacterial mobile genetic elements	BLAST+	https://github.com/clb21565/mobileOG-db	[135]
SARG 2.0	Antibiotic resistance gene database	ARGpore2, BLAST+, LAST	http://smile.hku.hk/SARGs	[130]
CARD	The comprehensive antibiotic resistance database	RGI, BLAST+	https://card.mcmaster.ca/	[129]
PHI	Pathogen–host interactions	BLAST+	http://www.phi-base.org/	[133]
VFDB	Virulence factor database	BLAST+	http://www.mgc.ac.cn/VFs/	[132]
BacMet	Antibacterial biocide and metal resistance genes	BLAST+	http://bacmet.biomedicine.gu.se/	[131]
ISFinder	Insertion sequences isolated from bacteria and archaea	BLAST+	https://isfinder.biotoul.fr/	[134]
SecReT6 v3	Type VI secretion system (T6SS)	BLAST+	https://bioinfo-mml.sjtu.edu.cn/SecReT6/	[136]
**Metabolism and elemental cycling databases**				
CAZY	Carbohydrate-active enZYmes database	BLAST+, HMMER, dbCAN3	http://www.cazy.org/	[137]
CYPED	Cytochrome P450 engineering database	BLAST+	http://www.cyped.uni-stuttgart.de	[138]
TCDB	Transporter classification system database	BLAST+	https://www.tcdb.org/	[139]
antiSMASH	Secondary metabolite BGCs	antiSMASH	https://antismash.secondarymetabolites.org/	[114]
Bigspace	Diversity of BGCs	Bigspace	https://bigscape-corason.secondarymetabolites.org/	[115]
NCycDB	Nitrogen cycle gene (sub)families	BLAST+, Diamond	https://github.com/qichao1984/Ncyc	[140]
SCycDB	Sulfur cycling genes and pathways	Diamond	https://github.com/qichao1984/SCycDB	[141]
MCycDB	Methane cycling genes	Diamond	https://github.com/qichao1984/MCycDB	[142]
PCyCDB	Phosphorus cycling genes	Diamond	https://github.com/ZengJiaxiong/Phosphorus-cycling-database	[143]
**Taxonomic databases**				
IMG/VR v4	Integrated microbial genome/virus system	BLAST+	https://img.jgi.doe.gov/vr	[144]
GTDB	Genome taxonomy database	GTDB-tk	https://gtdb.ecogenomic.org/	[145]
VirSorter2-DB	Diverse DNA and RNA virus genomes	VirSorter2	https://github.com/jiarong/VirSorter2	[146]
CheckV-DB	Complete viral genomes from metagenomes	CheckV	https://bitbucket.org/berkeleylab/CheckV	[147]
Kraken2-DB	Standard or custom RefSeq databases for taxonomic classification	Kraken2, Krakentools	https://benlangmead.github.io/aws-indexes/k2	[54]
Kaiju-DB	Taxonomic classification database includes Nr, RefSeq, progenomes, plasmid, and rvdb	Kaiju	https://bioinformatics-centre.github.io/kaiju/	[66]

*Note*: These databases are suitable for both general and long-read metagenomics. Considering the rapid advancements in this field, the content will be updated and maintained on GitHub (https://github.com/zhangtianyuan666/LongMetagenome) on a quarterly basis to uphold its usability and currency.

#### Public functional annotation databases

Public functional annotation databases provide researchers with insights into the metabolic capabilities and functional potential of genes found in metagenomic data. Key resources include the non-redundant protein database [119], the nucleotide database [120], and RefSeq [121], all curated by the NCBI. These databases offer species information along with functional annotations, aiding in the identification and classification of metagenomic sequences. Other commonly used databases for functional annotation include Gene Ontology (GO) [122], UniProt [123], Kyoto Encyclopedia of Genes and Genomes (KEGG) [124], and eggNOG [113]. These databases are frequently utilized to explore gene families, investigate gene functions, and analyze metabolic and regulatory pathways. Collectively, these databases offer a comprehensive understanding of the functional attributes of genes across a wide range of species. Rfam [125], Pfam [126], and TIGRfam [127] are databases that catalog and classify RNA and protein families based on hidden Markov models (HMMs). Pfam recognizes and describes protein families and domains, which is especially useful for annotating protein sequences and inferring their possible functions. Rfam is a database specializing in diverse non-coding RNA (ncRNA) families, encompassing rRNAs, transfer RNAs (tRNAs), small nuclear RNAs (snRNAs), microRNAs (miRNAs), and various other ncRNAs. TIGRfam is another database focusing on protein and RNA families primarily associated with microbial genomes. MBGD [128] is a comparative database of fully sequenced microbial genomes that helps with ortholog discovery, paralog grouping, motif analysis, and so on. These databases serve as indispensable resources for annotating metagenomic sequences and understanding the functions of microbial communities.

#### Resistance element and MGE databases

Several databases focusing on resistance elements and MGEs have been established to catalog ARGs, MGEs, and virulence factors (VFs) because of their critical roles in public health, epidemiology, and biotechnology. The comprehensive antibiotic resistance database (CARD) [129] and SARG [130] offer extensive information on ARGs. Additionally, the BacMet [131] database provides experimentally validated information on resistance mechanisms against metals and antibacterial biocides. The virulence factor database (VFDB) [132] meticulously organizes VFs from a wide range of bacterial pathogens, while PHI-base [133] compiles experimentally validated genes associated with pathogenicity, virulence, and other disease mechanisms, across diverse pathogens. This includes studies on host–pathogen interactions between hosts and infections and other disease mechanisms, extending beyond antibiotic resistance. ISFinder [134] and mobileOG-db [135] investigate MGEs in bacteria and archaea. These elements are essential for the dissemination of ARGs and genomic rearrangement, emphasizing their significance in microbial evolution and adaptability. Additionally, the SecReT6 [136] database offers comprehensive information on bacterial type VI secretion systems (T6SSs), which mediate complex interactions between bacteria and eukaryotes. Together, these databases provide valuable insights into the dissemination of antibiotic resistance and pathogenic capabilities of microbial communities.

#### Metabolism and elemental cycling

Metabolism and elemental cycling databases deal with the study of enzymes and pathways involved in these processes. The CAZy database [137], CYPED [138], and TCDB [139] provide extensive resources focusing on genes related to metabolism. The CAZy database is particularly valuable for understanding enzymes involved in carbohydrate degradation, modification, and biosynthesis. CYPED classifies cytochrome P450 enzymes involved in oxidative metabolism, while TCDB organizes transporters based on their evolutionary relationships and functional roles. The antiSMASH database offers a curated collection of BGCs, while Big-SCAPE [115] categorizes these clusters into groups based on similarity, generating a network for large-scale investigation of gene clusters associated with natural products. The NCycDB [140], SCycDB [141], MCycDB [142], and PCyCDB [143] databases provide tailored insights into specific metabolic pathways, allowing for detailed exploration of metabolic functions and processes.

#### Taxonomic databases

Taxonomic databases provide essential information on the classification and taxonomy of organisms. These databases are used to assign taxonomic labels to sequencing reads and infer the taxonomic composition of microbial communities. The IMG/VR [144] database is designed for analyzing and evaluating publicly available genomes of bacteria, archaea, and viruses. The database includes metadata, functional annotations, and taxonomic classifications. The GTDB offers a standardized and reliable classification system for bacteria and archaea based on genome sequences [145]. It utilizes a phylogenetic approach to categorize organisms, ensuring a more accurate and up-to-date classification. The VirSorter2 [146] database is associated with the VirSorter pipeline, which is used to detect and categorize viral sequences in metagenomic data. The CheckV [147] database collects lineage-specific marker genes to assess the quality, completeness, and taxonomy of viral genomes obtained from metagenomes. Kraken2DB [54] and KaijuDB [66] are widely used for assigning taxonomic labels to metagenomic sequences. They utilize both publicly available databases and customizable features to classify taxa based on research requirements. Long-read shotgun metagenomics studies rely on these databases to categorize organisms at various taxonomic levels, from species to phyla. This taxonomic information assists researchers in clarifying the composition, structure, and ecological functions of microbial communities within their respective ecosystems.

### Applications of R packages for visualization in long-read metagenomics studies

Visualization plays a pivotal role in exploring, analyzing, and communicating complex biological data, particularly in the context of long-read sequencing technology used in metagenomics. Several R packages can effectively process and visualize long-read metagenomic data (**Table 3**). It is important to note that these R packages are also suitable for comprehensive short-read metagenomic analysis, making them equally potent in the realm of long-read metagenomics.

**Table 3:** Applications of R packages for visualization in metagenomics studies

Application	Package
Metagenome and microbiome analysis & visualization	MetagenomeSeq [148], EasyAmplicon [149,150], EasyMetagenome [151], EasyMicrobiome [152], MicrobiomeStat [153], microbiome [154], EasyMicroPlot [155], Phyloseq [156]
Data visualization & plotting	ImageGP [157], clusterProfiler [158], igraph, Compositions [159], MicrobiomeStatPlots [160], Corrplot [161]
Multi-omics	ivTerm [162], mixOmics [163]
Data processing & statistical analysis	ggplot2 [164], ggtree [165], networkD3 (https://christophergandrud.github.io/networkD3), circlize [166], ggvenn, ggmap [167], ggpubr, UpSetR [168], Pheatmap

*Note*: These databases are equally suitable for both general and long-read metagenomics. Considering the rapid advancements in this field, the content will be updated and maintained on GitHub (https://github.com/zhangtianyuan666/LongMetagenome) on a quarterly basis to uphold its usability and currency.

These packages can be classified into four categories: (1) Analysis and visualization of metagenomes and microbiomes: packages such as MetagenomeSeq [148], EasyAmplicon [149,150], EasyMetagenome [151], EasyMicrobiome [152], MicrobiomeStat [153], microbiome [154], EasyMicroPlot [155], and Phyloseq [156] are designed for importing, analyzing, statistically processing, and visualizing microbiomic data. These packages facilitate the understanding of biodiversity and microbial ecosystem functions. (2) Data visualization and plotting: packages like ImageGP [157], clusterProfiler [158], igraph (https://github.com/igraph/igraph), compositions [159], MicrobiomeStatPlots [160], and Corrplot [161] provide a range of analysis and visualization options, from simple charts to intricate networks, clustering, and dimensionality reduction studies. (3) Multi-omics analysis: tools including ivTerm [162] and mixOmics [163] combine and analyze data from several biological layers, such as genomics, transcriptomics, and proteomics. These tools are essential for clarifying relationships within complex biological systems. (4) Data processing and statistical analysis: widely used packages such as ggplot2 [164] and its extensions (*e*.*g*., ggtree [165]), along with networkD3 (https://christophergandrud.github.io/networkD3), circlize [166], ggvenn (https://github.com/yanlinlin82/ggvenn), ggmap [167], ggpubr (https://github.com/cran/ggpubr), UpSetR [168], and Pheatmap (https://github.com/raivokolde/pheatmap), offer a wide range of data processing and visualization options from basic to advanced. These tools empower researchers to create customized visuals that effectively explain the results of their data analysis. The precise objective of each R package is outlined in Table S2. Overall, these R packages significantly enhance the complexity and depth of metagenomic analysis, enabling researchers to drive meaningful biological insights from large and multifaceted datasets.

## Summary and outlook

In the field of metagenomics, advanced sequencing technologies, such as ONT and PacBio, have greatly transformed the study of complex microbial communities. These technologies offer long-read sequencing that can cover entire microbial genomes, overcoming the limitations of short-read sequencing. These advancements provide new opportunities to resolve genomic repeat regions, identify SVs, and accurately characterize uncultivated microorganisms. This review focuses on the computational tools and resources that leverage ONT and PacBio technologies in metagenomics. While these sequencing techniques yield extensive metagenomic data, retrieving valuable information and understanding the structure and function of microbial communities require robust computational tools and resources.

### Breakthrough

A number of specialized software tools have been developed to address the distinct challenges posed by long-read metagenomic data. For *de novo* assembly, tools such as metaFlye and Lathe for ONT and HiFiasm-meta and metaMDBG for PacBio are widely used. Basecalling tools such as Dorado (for ONT) and SMRTlink (for native PacBio) enhance the precision of raw sequence data. Metagenomic binning tools, such as BASALT and GraphMB, utilize long-read data to achieve a more precise resolution of individual species within a community. Advancements in taxonomic classification lead to the development of specialized classifiers, such as BugSeq2 and Metamaps, specifically designed for long-read metagenomes.

### Limitation

Despite these advancements, there remain areas where long-read applications require further development or optimization. Nevertheless, the effectiveness and features of these software tools have not been thoroughly validated, with most evaluations still relying heavily on short-read data [169]. We anticipate that as long-read technologies continue to evolve, these approaches will yield more effective outcomes in future metagenomics studies. There is a need for integrated pipelines that optimize the transition from unprocessed data to biological insights. These pipelines should encompass quality control, assembly, binning, and functional annotation. Additionally, the development of innovative statistical techniques, machine learning, and artificial intelligence algorithms will be crucial for handling the noise and inherent biases in long-read data analysis.

Specifically, resources for metagenomic methylation and metaPore-C are limited. In metagenomics, identifying methylation sites and functionally annotating complex communities are essential for understanding the methylation patterns of microbes in environmental samples. Metagenomic methylation research can benefit from long-read sequencing technology, but specialized methylation analysis techniques are required to analyze methylation sites. metaPore-C improves assembly and binning, linking plasmids, hosts, and viruses. Currently, ONT is the only entity that provides experimental techniques and strategies for metaPore-C. However, its specific applications have yet to be reported.

### Future

By 2025, the sequencing throughput of PacBio is expected to witness a remarkable increase, allowing for faster and more comprehensive coverage of large metagenomic studies. Concurrently, ONT is expected to achieve a substantial improvement in read accuracy, targeting Q20 or even potentially reaching Q30. This level of accuracy would bring the error rate of ONT much closer to that of traditional short-read methods, thereby increasing confidence in metagenomic assembly. Such advancements will expand the role of ONT in demanding applications, such as metagenomics, where high accuracy is critical for identifying low-abundance species and discerning between closely related organisms. With these advancements, researchers will gain unprecedented insights into the large-scale genetic variants and epigenetic patterns across diverse populations of microbes, viruses, and host organisms, contributing to a deeper understanding of the complex interactions between genetics and the environment in health and disease.

In summary, although notable advancements have been made in developing computational tools and resources for metagenomics using ONT and PacBio data, continuous innovation and adaptation remain crucial for fully exploiting the capabilities of these powerful sequencing technologies to understand complex microbial ecosystems.

Box 1 Key technical terms **Flow cell:** A flow cell is a device used in high-throughput sequencing systems. It consists of a glass slide with nanowells or channels for processing nucleic acid samples. Flow cell chemistry involves fluorescently labeled nucleotides, DNA polymerase, and buffer systems. Precise control of these processes is crucial for accurate sequencing data.

**Quality score:** A quality score (Q score) measures DNA sequencing base call accuracy, with the score inversely proportional to the error rate. For example, a Q score of 20 (Q20) corresponds to an error probability of 1 in 100 (99% accuracy), while Q30 corresponds to an error probability of 1 in 1000 (99.9% accuracy).

**MAG:** An MAG is a genome that is reconstructed from metagenomic sequencing data, which involves the collective analysis of genetic material recovered directly from environmental samples. MAGs are obtained through bioinformatics techniques that bin and assemble sequences from complex microbial communities.

**Binning:** In metagenomics, binning is a bioinformatics process that groups DNA sequences into discrete bins, each representing a putative genome.

*Note*: MAG, metagenome-assembled genome.

## Supplementary Material

qzaf075_Supplementary_Data

## Data Availability

The list of all software, along with introduction to R packages, installation instructions, and usages methods for the noteworthy software, has been uploaded to GitHub (https://github.com/zhangtianyuan666/LongMetagenome). The list of software is updated every three months to ensure that users have access to the latest developments.

## CRediT author statement

**Tianyuan Zhang:** Conceptualization, Writing – original draft, Visualization, Writing – review & editing. **Mian Jiang:** Recourses, Writing – review & editing. **Hanzhou Li:** Writing – review & editing. **Yunyun Gao:** Visualization, Writing – review & editing. **Salsabeel Yousuf:** Writing – review & editing. **Kaimin Yu:** Visualization. **Xinxin Yi:** Visualization. **Jun Wang:** Writing – review & editing. **Lulu Yang:** Supervision, Writing – review & editing. **Yong-Xin Liu:** Conceptualization, Supervision, Funding acquisition, Writing – review & editing. All authors have read and approved the final manuscript.
