# Supplementary material for: Computational Tools and Resources for Long-read Metagenomic Sequencing Using Nanopore and PacBio
Source: Genomics Proteomics Bioinformatics. 2025 Aug 22;23(4):qzaf075. doi: 10.1093/gpbjnl/qzaf075 (PMC12631790; doi:10.1093/gpbjnl/qzaf075)
Supplement: qzaf075_Supplementary_Data [file qzaf075_supplementary_data.zip › Supplementary Material Captions.docx]

**Supplementary material**

**Table S1 The software in metagenomics studies**

**Table S2 The description of visualizing R packages in metagenomics studies**
